# Experimental and Numerical Study on Hysteretic Behavior of Frictional Energy Dissipation Steel Truss

**DOI:** 10.3390/ma16186273

**Published:** 2023-09-19

**Authors:** Zhibin Zhou, Xuhong Zhou, Qishi Zhou, Huawei Fu, Shuaishuai Liu

**Affiliations:** 1 School of Civil Engineering, Central South University, Changsha 410075, China; zzbbridge@csu.edu.cn (Z.Z.);; 2 National Engineering Research Center of High-Speed Railway Construction Technology, Changsha 410075, China; 3 Engineering Technology Research Center for Prefabricated Construction Industrialization of Hunan Province, Changsha 410075, China; 4 School of Civil Engineering, Chongqing University, Chongqing 400044, China

**Keywords:** staggered truss framing systems, friction damper, hysteretic performance, hysteretic test, finite element analysis

## Abstract

In this study, the hysteretic behavior of a novel frictional energy dissipation steel truss (FED-ST) is examined. The proposed FED-ST incorporates a friction damper with brass as the friction material into the top chord of traditional truss to improve the seismic performance of the staggered truss framing systems. A FED-ST specimen with a scale of 1:2.5 was subjected to a hysteresis test. The hysteretic behavior, ductility, and energy dissipation capability were analyzed considering the test findings. It is demonstrated that the FED-ST specimen has favorable ductility and an energy dissipation capacity that is 7.3 times more than that of a conventional truss specimen. The test findings were then used to compare and validate a finite element (FE) model. The FE analysis results are in strong agreement with the test results, demonstrating the validity of the modeling approach. To further investigate the impact of the cover plate width on the behavior of the FED-ST, preliminary parametric research was also carried out.

## 1. Introduction

**Figure 1:**
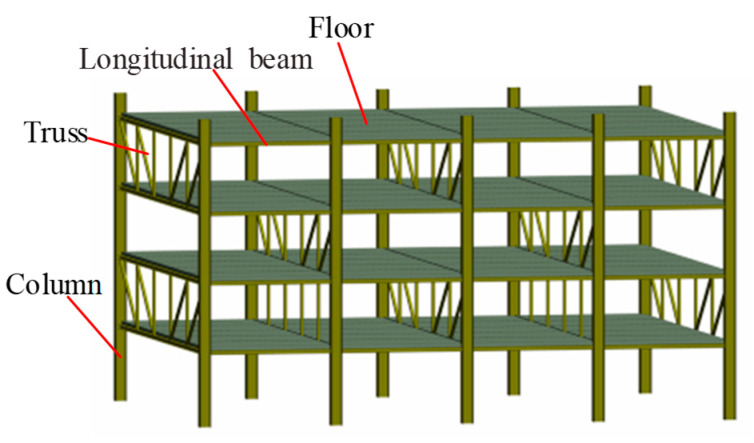
Structure diagram of the STFS.

The staggered truss framing system (STFS) is primarily utilized in residential structures like flats, dorms, hotels, etc. [1]. It is made up of columns, longitudinal beams, floors, and trusses (Figure 1). STFS contains many truss members and the bending moment of the columns under lateral loads is very small. Therefore, compared with steel moment frame systems, it can save more on consumption of steel and it is a very efficient structural system.

Numerous studies have been done on the static and dynamic performance and design solutions of STFS since the 1960s [2]. A 1:8 scaled STFS specimen’s performance under low cyclic loading was studied by Zhou et al. [3], and some ideas for the design of the STFS were put forth according to the outcomes of tests and FEA. A 6-story STFS specimen built without a fire-proof coating and subjected to a pool fire was researched by Chen et al. [4] to determine its mechanical behavior and manner of failure. Kim et al. [5,6] evaluated the seismic performance of STFS, and developed a seismic design strategy for friction dampers within STFS. Zhou et al. [7] designed and fabricated two 1:2.5 scaled truss specimens and carried out tests under cycle load. The results of the test indicate that the ductility of truss specimens is worse than that of the ordinary moment-frame system. A 14-story STFS model with a size of 1:8 was subjected to a number of pseudo-dynamic tests by Zhou et al. [8], and the seismic performance of the test model was examined considering the test findings. Zhou et al. [9] calculated the demand for hybrid STFS under earthquake through numerical simulation and revealed damage-control behavior. However, STFS is not listed as a fundamental seismic-force-resisting system in mainstream specifications [10], which suggests that more work should be done to improve the system’s seismic performance.

Because of their durability and affordability, frictional dampers have been frequently used in structural engineering in the past few decades. Boggian et al. [11] proposed a retrofitting solution for RC buildings based on the combined use of cross-laminated timber (CLT) panels and asymmetric friction connections (AFCs). The research showed that the energy dissipation of the AFC increases the structural dissipation capacity and reduces the displacement demand. Qiu et al. [12] investigated the cyclic behavior of a steel self-centering (SC) rocking column. The steel SC rocking column consists of an H-section steel column and a pair of shape memory alloy (SMA) slip-friction dampers (SMASFDs). It was found that up to a drift ratio of 7%, the rocking column maintained stable flag-shaped hysteresis, which was characterized by excellent SC capability and satisfactory damping capacity. The experimental research by Zhang et al. [13] reveals that compared with steel-stainless steel friction pairs, the friction behavior of steel-brass pairs is more stable, repeatable, and predictable. Moreover, the material wear is also slighter, highlighting their advantages as friction pairs. According to research by Kim et al. [14] on a 3D-office building frame, a limited number of BRBs (Buckling-Restrained Braces) could enhance the seismic capacity of the structure by minimizing story drift. Using two numerical models, Bruschi et al. [15] tested a novel friction damper used in existing building retrofitting. According to the study’s findings, the friction damper is advantageous for dispersing seismic energy, preventing the frame’s lateral deformation, and simultaneously managing a rise in internal forces and structural acceleration. An inelastic moment frame was proposed to have its seismic vibration controlled using rotating friction dampers (RFDs) by Jarrahi et al. [16]. The frame outfitted with the optimal RFD was subsequently evaluated and put to the test against four historical earthquake records. The findings show that it performs better seismically than the uncontrolled structure. A new self-centering damper was developed by Veismoradi et al. [17] and a sample prototype was created for testing. The test findings demonstrate the damper’s capacity for energy dissipation and reliable self-centering function that does not require any post-event maintenance. A brand-new kind of shape memory alloy self-centering friction damper was created by Li et al. [18]. When used with earthquake-resistant structures, its self-centering capability and inexpensive maintenance costs after an earthquake provide it a significant benefit. The effectiveness of the suggested friction damper with double slip loads (DSL) under moderate and strong seismic excitations was examined by Ghorbani et al. [19]. In comparison to the more traditional dampers, the results show that the suggested DSL dampers are more successful at lowering the structural seismic response.

To improve the seismic performance of STFS, the authors developed a new frictional energy dissipation steel truss (FED-ST). The difference between FED-ST and conventional trusses is that it integrates a friction damper in the top chord. In previous studies [20], the authors conducted mechanism analysis and experimental study on a FED-ST specimen with a sliding distance of ±18 mm. To further investigate the seismic behavior of FED-ST with a long sliding distance, a 1:2.5 scaled specimen with a sliding distance of ±40 mm was made for the cyclic load test in this paper. In addition, numerical simulations of FED-ST were conducted using the commercial finite element software ABAQUS 6.14 and compared with experimental results. 

## 2. Configuration of FED-ST

**Figure 2:**
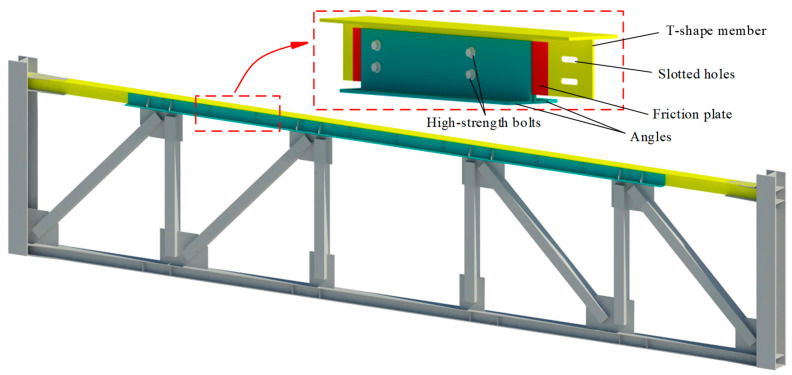
Configuration of the FED-ST system.

The configuration of FED-ST is seen in Figure 2, where the top chord consists of a T-shaped member with long slot holes, two angle members, and two brass friction plates. The T-shaped member, brass friction plates, and angle members are connected through a series of bolts, and the brass friction plates can make the friction between the angle members and the T-shaped member smoother. Energy is dissipated through the friction generated between brass plates and steel plate, and the effective energy dissipation has been verified by many studies [21,22]. Sliding and friction will occur between the angle members and the T-shaped member to dissipate energy when the truss structure undergoes rare earthquakes.

**Figure 3:**
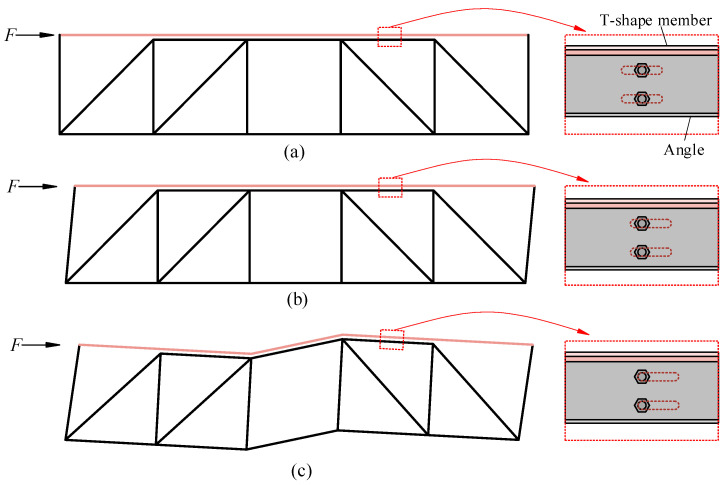
Schematic diagram of three stages of FED-ST (**a**) Elastic stage, (**b**) Friction stage, (**c**) Plastic stage.

The behavior of FED-ST under earthquakes is seen in Figure 3, which consists of three stages. FED-ST is in the elastic stage under frequent earthquakes, just like a traditional truss. At this time, the lateral load does not reach the activation force of the frictional chord damper (maximum static friction force generated by the bolts). 

When the lateral force exceeds the activation force of the frictional chord damper, sliding occurs between the T-shaped member and the angle members, which is the friction stage. Most members of the truss remain elastic, except for the column-chord joints.

As the sliding distance continues to increase, when the high-strength bolts contact the end of the long slot holes, the relative movement between them reaches its maximum and cannot further slide. As the force continues, FED-ST is about to yield and enter the plastic stage.

## 3. Experimental Program

### 3.1. Design of Specimen

**Figure 4:**
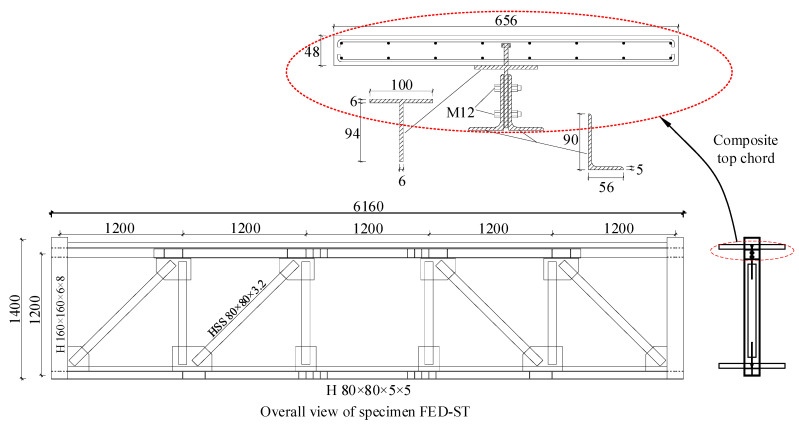
Dimensions of specimen FED-ST (mm).

A 5-panel FED-ST was designed according to relevant codes [23,24]; the length of the truss is 15 m, and the height is 3 m. Then, a 1:2.5 scale specimen FED-ST40 was fabricated and tested. The dimensions of FED-ST40 are shown in Figure 4. The floor slabs are connected to the chords through a number of Φ8 mm studs, with a spacing of 120 mm between studs. HSS (hollow structural section members) were used to make webs, H-shaped members were used to make the bottom chord, and the top chord is a frictional chord. The friction plate between T-shaped member and angle members is made of brass with a thickness of 1 mm. The slot holes’ length $L_{s}$ is 92 mm, therefore, the sliding range $\Delta_{f}=(L_{s}-d_{b})/2$ is ±40 mm, where $d_{b}$ is the diameter of the bolts. There are 22 high-strength bolts with a diameter of 12 mm arranged in the frictional chord in double rows; the pre-tightening force of each bolt is 20 kN. The tightening force in bolt was controlled by the torque applied according to Equation (1) from the Chinese standard for the design of steel structures [25]:

(1) T=kPd

where *T* is the applied torque, *k* is a constant, which is 0.13 for all the bolts in this study as provided by the manufacturer, *P* is the tightening force in bolt, and *d* is the bolt diameter. In this study, applied torque *T* for every bolt in specimen FED-ST40 is 31.2 N·m.

The chords and webs of the FED-ST specimen were made of Q355 steel. The concrete floors were made of C30-grade concrete. According to the standard [26], compressive tests were conducted on six concrete cube specimens with an edge length of 150 mm to calculate the material properties of the concrete cubes. Table 1 shows the material properties. The cubic characteristic compressive strength was taken as $f_{\mathrm{cu},k}=f_{\mathrm{cu}}-1.645\sigma_{\mathrm{cu}}$ by the Chinese code [27], where the $f_{\mathrm{cu}}$ is the cubic measured average compressive strength, the $\sigma_{\mathrm{cu}}$ is the standard deviation. 

### 3.2. Experiment Setup

**Figure 5:**
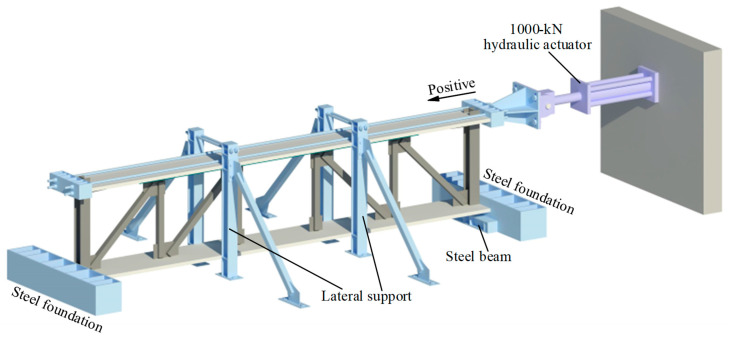
Experiment setup of specimen FED-ST40.

In Figure 5, the experiment setup is depicted. To prevent any movement, the steel foundation and steel beam are attached to the stiff floor using several anchor bolts. The bottom surface of the column was connected to a steel beam fixed on the rigid floor to prevent the vertical movement of the specimen. The steel foundation at both ends of the specimen was used to prevent horizontal movement of the bottom of the specimen. The lateral load was applied by a 100-ton servo-hydraulic actuator fixed on the reaction wall. And there were two loading beams at both ends of the top of the specimen, which were connected by four large-diameter fine-rolled threaded steel bars. Two lateral supports were set on both sides of the specimen, which were fixed on the rigid floor to prevent the specimen from lateral collapse during the test. In addition, some round steel rods were placed between the concrete slabs and the lateral supports to reduce the frictional resistance during testing.

**Figure 6:**
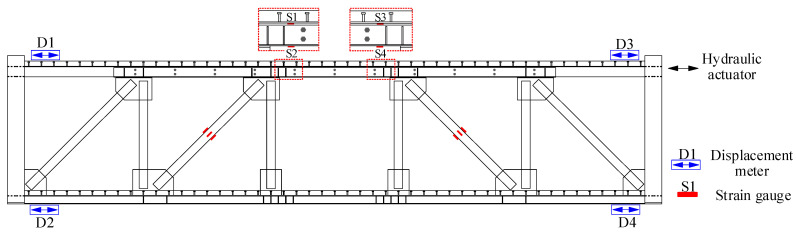
Measuring arrangements for the specimen.

During the experiment, the force applied to the specimen was measured using force sensors embedded in the actuator, and the deformation and strain were measured using displacement meters and strain gauges. The arrangement of the sensors is shown in Figure 6. D1–D4 represents displacement meters arranged at the four corners of the specimen to measure the displacement at the top and bottom floor slabs on both ends of the specimen. During the experiment, the left-end inter-story drift is defined as UL = D1–D2, the right-end inter-story drift is defined as UR = D3–D4, and the final inter-story drift is defined as U = (UL + UR)/2.

The test load is applied by inter-story drift ratio, with 2 cycles at each level in 0.25%, 0.5%, 0.75%, 1%, 1.5%, 2%, 2.5%, 3%, 3.5%, 4%, …. The loading displacement increases by 1% each time after 4% until the specimen fails.

## 4. Test Results and Discussion

### 4.1. Failure Characteristics

When the inter-story drift ratio was 0.25% (±3 mm), the lateral force did not reach the activation force of the frictional chord damper, and the specimen was in the elastic stage. 

**Figure 7:**
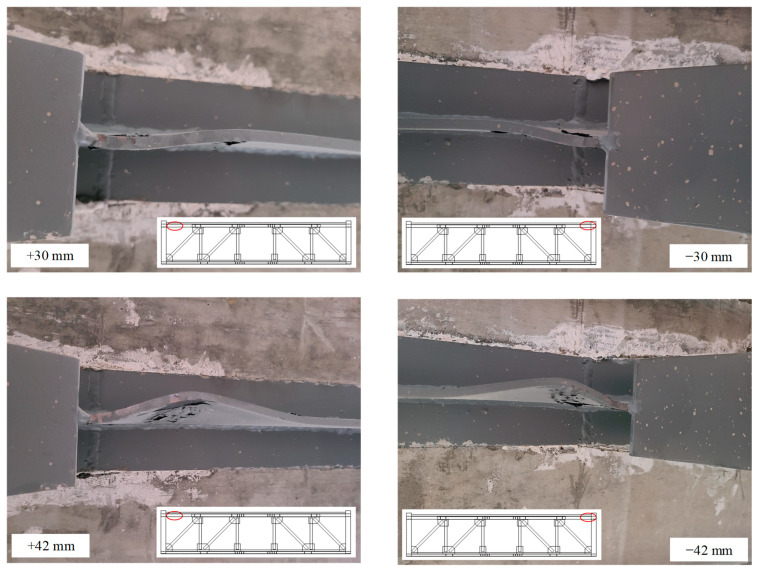
Deformation at column-chord joint.

**Figure 8:**
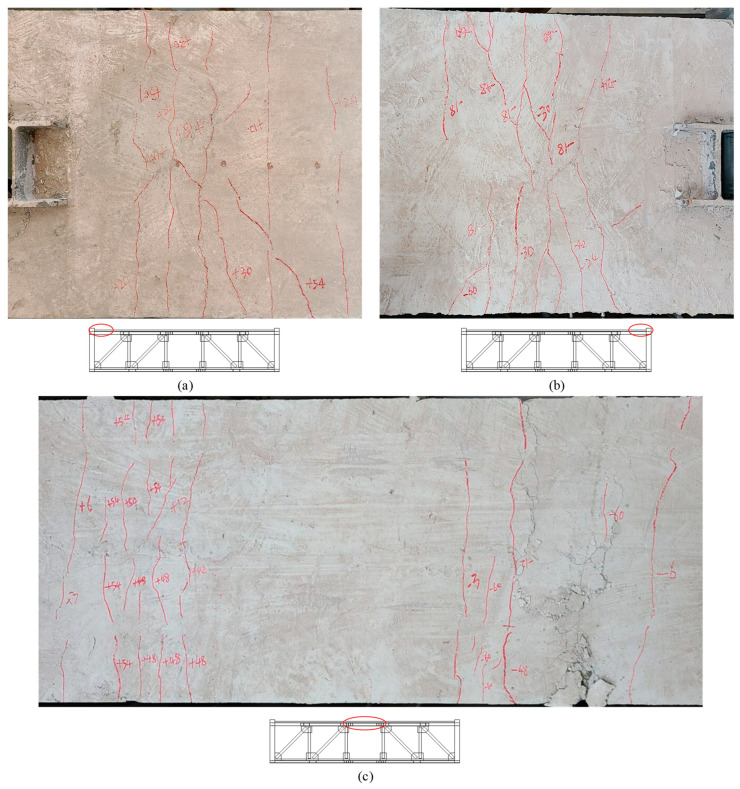
The distribution of cracks in the floor slabs (**a**)The end of the truss away from the hydraulic actuator, (**b**)The end of the truss near the hydraulic actuator, (**c**)Vierendeel panel.

As the test continued, the specimen emitted a sound of metal friction, and the specimen entered the friction stage. When the inter-story drift ratio was 2.5% (30 mm), the compression web of the T-shaped member at the column-chord joint buckled slightly, and the buckling increased when the displacement reached ±42 mm (Figure 7). At the same time, the cracks in the floor above the T-shaped member increased (Figure 8a,b), and plastic hinges have formed here. However, the strain measurement results indicated that the diagonal webs and the vierendeel panel chords remained in an elastic state.

**Figure 9:**
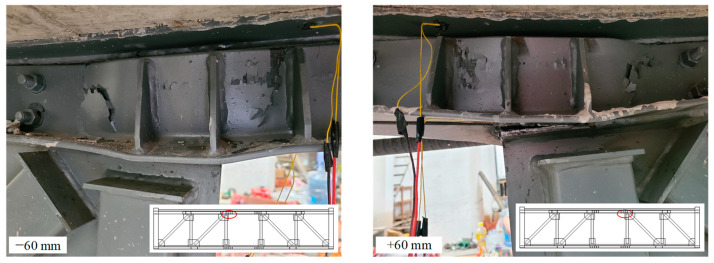
Deformation at vierendeel panel.

When the displacement exceeded 42 mm, the specimen no longer emitted frictional sound, and the force continued to increase, indicating that the frictional chord had stopped sliding, and the specimen entered the plastic stage. As the specimen continued to load, cracks in the vierendeel-panel slabs increased, and the angle members at the end of the vierendeel panel began to exhibit slight buckling. When the inter-story drift ratio was 5% (60 mm), as shown in Figure 8c, the vierendeel-panel concrete floors were seriously damaged, and the out-of-plane buckling of the angle members was more pronounced (Figure 9). In the second cycle of ±60 mm, due to the buckling of the angles and damage of the floor slabs, the force of actuator was 388 kN, which is 77% of the peak load, and the truss failed. Besides that, the measurement results of strain indicated that the strain of the webs had not exceeded the yield strength.

The experiment verified that the FED-ST showed the expected failure mode and expected three-stage behavior under lateral forces.

**Figure 10:**
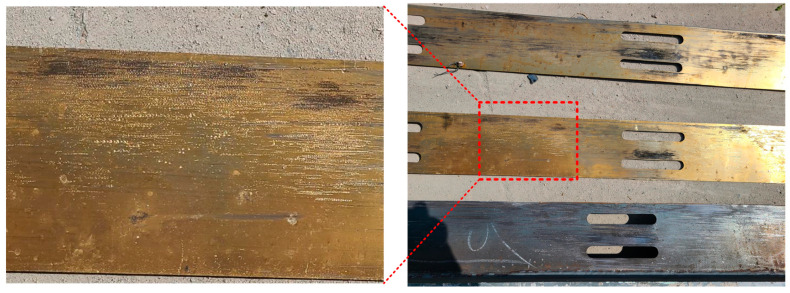
The friction scratches on the T-shape steel and the brass plates.

After the experiment, the frictional chord was disassembled and friction scratches were observed on both the T-shape steel and the brass plates. As the brass is softer than the steel, the scratches on the brass plates are more obvious, as shown in Figure 10.

### 4.2. Hysteretic Responses

**Figure 11:**
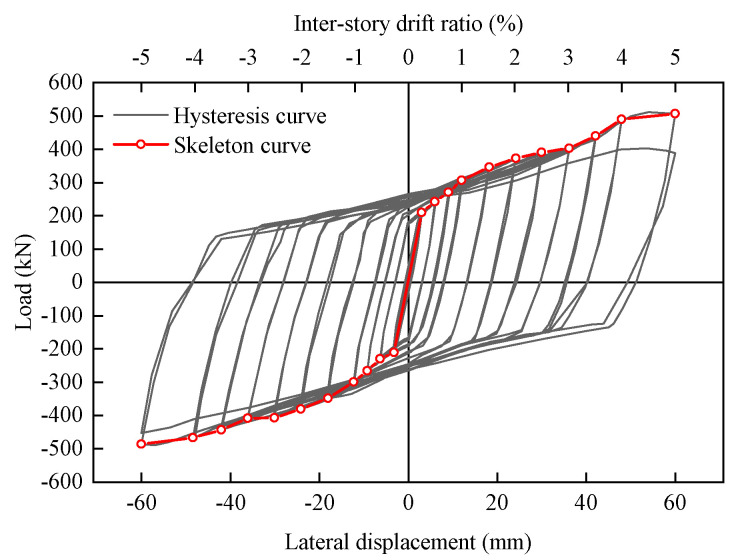
Hysteresis curves and skeleton curves of the specimen FED-ST40.

Figure 11 shows the hysteresis curve and skeleton curve of the specimen FED-ST40. Compared with the conventional truss specimen ST1 previously studied by the author [7], the ultimate displacement of FED-ST40 is 60 mm, 2.6 times that of the conventional truss specimen (22.7 mm). In addition, due to the effect of the frictional chord, the area of the hysteresis loop of FED-ST40 is larger than that of ST1 [7].

The capacity of structural plastic deformation under lateral loads is often measured using ductility, the ductility coefficient *μ* defined as:

(2) $$\mu =\Delta_{u}/\Delta_{y}$$

There is an obvious inflection point at 3 mm in the skeleton curve of the FED-ST40, which can be considered as the yield point $\Delta_{y}$ (mm). The ultimate displacement $\Delta_{u}$ (mm) generally refers to the displacement when the load is reduced to 85% of the peak load. Due to the failure of specimen FED-ST40 at a loading displacement of 60 mm, 60 mm is taken as the $\Delta_{u}$ of FED-ST40, which is significantly higher than that of the conventional truss specimen (22.7 mm) [7] and which meets the requirements of mainstream specifications [28,29] for inter-story drift of steel structures (0.02 rad). The ductility coefficient of FED-ST40 is 20, which is much larger than that of a conventional truss specimen (2.91) [7], indicating that the FED-ST has good ductility.

### 4.3. Energy Dissipation Capability

**Figure 12:**
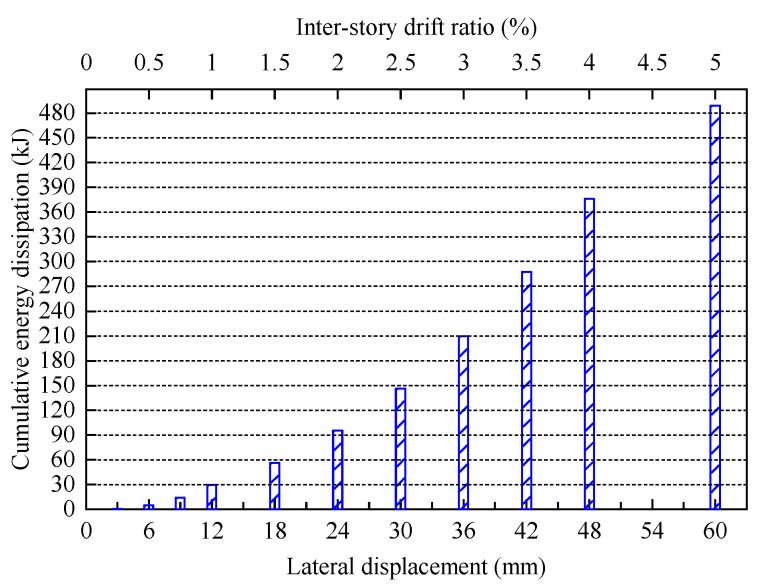
The cumulative energy dissipation of FED-ST40.

The cumulative energy dissipation of FED-ST40 is shown in Figure 12. The specimen FED-ST40 was in the elastic stage when the displacement was 3 mm, with little energy dissipation. After the displacement reached 6 mm, FED-ST40 had mainly dissipated energy through friction, and the energy dissipation had significantly increased. When the displacement was 42 mm, the cumulative energy dissipation reached 287.6 kJ, the sliding displacement of the specimen got to the maximum value, then the chords were about to enter a plasticity state. When further loaded to 60 mm, the specimen failed. The total energy dissipation is 7.3 times that of the conventional truss specimen [7]. The above results indicate that the energy dissipation capability of FED-ST is good.

**Figure 13:**
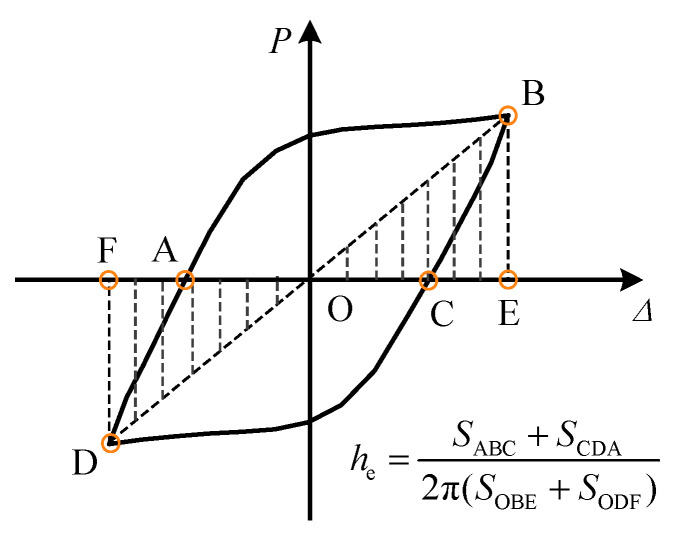
Equivalent viscous damping coefficient $h_{e}$.

**Figure 14:**
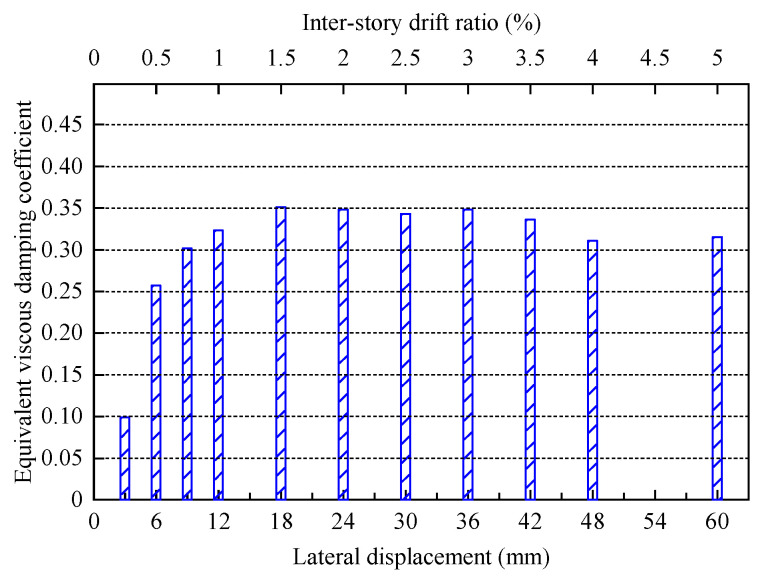
The equivalent viscous damping coefficient of FED-ST40.

The equivalent viscous damping coefficient $h_{e}$ can be used to measure the energy dissipation capacity of a structure, defined as $h_{e}=(S_{\mathrm{ABC}}+S_{\mathrm{CDA}})/2\pi (S_{\mathrm{OBE}}+S_{\mathrm{ODF}})$, where $S_{\mathrm{ABC}}$, $S_{\mathrm{CDA}}$, $S_{\mathrm{OBE}}$, and $S_{\mathrm{ODF}}$ are shown in Figure 13. The variation of the $h_{e}$ of the specimen with lateral displacement is shown in Figure 14. After the frictional chord damper is activated (6 mm–60 mm), the $h_{e}$ of FED-ST40 remains high (0.25–0.35), showing the excellent capability of energy dissipation.

According to the previous studies, the FED-ST has good energy dissipation capability which will greatly improve the seismic performance of the STFS.

## 5. Numerical Simulation

### 5.1. Overview

**Figure 15:**
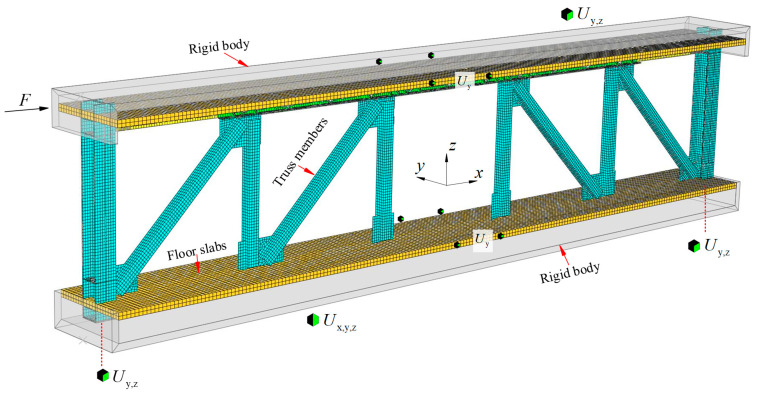
FE model of the FED-ST40.

The finite element model (FEM) was built using the commercial element program ABAQUS 6.14 for additional numerical simulation investigation. A nonlinear FE model FE-test was built based on the FED-ST40 hysteresis test in order to validate the accuracy of the modeling approach and materials constitutive model. The FEM’s geometrical dimension and boundary condition were following that utilized in the experimental study for the specimen, as shown in Figure 15. All component connection settings are consistent with the experiment, such as using “tie” for chord-column connection, which can be considered as a rigid connection, and “tie” is also used for web-chord connection. To simulate the loading pattern of the experiment, a rigid body was set up at the height of the top and bottom floor slabs of the specimen. The rigid body at the bottom floor was constrained by displacement in three directions of XYZ to simulate the foundation, while the rigid body at the top floor was constrained by displacement in two directions of YZ, and the load was applied in the X direction.

Considering the computing costs and the accuracy of results, the shell element S4R was used to simulate the web members, while the other components were simulated using the solid element C3D8R. The reinforcing bars and head studs are modeled using truss elements (T3D2), and assuming it is embedded in the concrete floor slabs. The meshes in the plastic area have been refined to a size of approximately 5 mm, while in the rest zones, 20 mm or smaller was the largest element size allowed. The “surface-to-surface contact” was created between the contact pieces. The tangential behavior of the contact property setting was set to “penalty” and the frictional coefficient was entered. The normal behavior was set to “hard” contact. The frictional coefficient was calibrated to 0.25 for the brass-steel contacting surfaces and was tuned to 0.5 for the concrete-steel contacting surfaces [30,31]. the expected slip force *f* can be calculated based on Coulomb friction theory:

(3) f=nμrN

where *n* is the number of high-strength bolts applying the pressure, which is 22 in this study; *μ* is the friction coefficient, calibrated as 0.25 at the steel–brass interface; *r* is the number of friction surfaces, which is 2 in this study; *N* is the bolt pre-tightening force, which is 20 kN in this study. According to Equation (3), the expected slip force is 220 kN.

The pre tightening force of a single bolt in the finite element model is consistent with the test, set at 20 kN, and the pre-load of the bolt is applied using the temperature method. Set the linear expansion coefficient in the bolt material parameters, and then establish a cooling load step on the bolt before the formal loading step of the test. By attempting to determine the cooling temperature, a pre-load of 20 kN was generated on the cooled bolts.

### 5.2. Materials Models

#### 5.2.1. Concrete

The stress–strain relationship of concrete is determined based on previous material properties tests and Reference [32]. The elastic modulus ($E_{c}$) and Poisson’s ratio ($\nu_{c}$) are set to 9500 $f_{\mathrm{cu}}{}^{1/3}N/{\mathrm{mm}}^{2}$ and 0.2, respectively, where $f_{\mathrm{cu}}$ is the cubic compressive strength of concrete, which is taken as 34.8 MPa according to the material properties tests.

The FE modeling for concrete uses the damage plasticity model in ABAQUS. According to the Reference [33], the pertinent parameters utilized for this material model are specified. Table 2 displayed the key parameter values for concrete. Based on ABAQUS, the unloading modulus was specified as $\left(1-D_{1}\right)E_{c}$, where $D_{1}$, which may be expressed as follows, is the elastic modulus’ damage variable:

(4) $$D_{1}=1-{\left(\frac{\sigma^{2}}{2E_{c}W}\right)}^{\frac{t}{t+1}}$$

where, *W* is the total strain energy density, defined as $\int_{0}^{\varepsilon }\sigma (\varepsilon )d\varepsilon$; *t* is defined according to the data fitting with the value of $t=\frac{1+0.05{\left(\varepsilon /\varepsilon_{0}\right)}^{4}}{3+0.05{\left(\varepsilon /\varepsilon_{0}\right)}^{4}}$ and $\varepsilon_{0}$ is the peak strain of concrete under compression and tension. The units of *W*, $E_{c}$, and σ(ε) in the Equation (4) should be unified.

#### 5.2.2. Steel

The combined hardening model in ABAQUS is adopted when modeling the constitutive behavior of steel using an elastic-plastic approach. Related parameters in ABAQUS are shown in Table 3 [34]. The yield strength $f_{y}$ is 407 MPa based on the material properties tests.

### 5.3. Validation of the Finite Element Model

**Figure 16:**
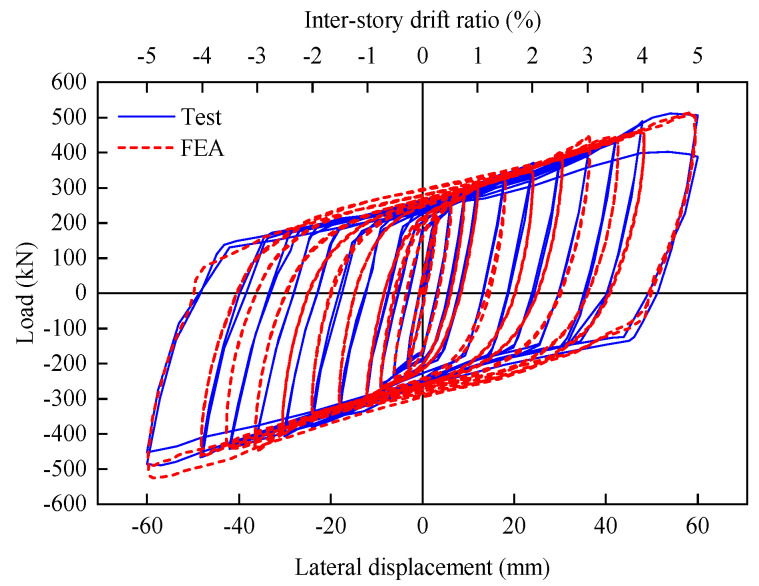
Test and FEA of hysteretic curves for FED-ST40.

Figure 16 contrasts the hysteretic curves between the specimen’s numerical simulation and experiment. The main characteristics of the experimental hysteretic curves were properly reflected by the numerical model, including the initial lateral stiffness, and the peak strength. 

**Figure 17:**
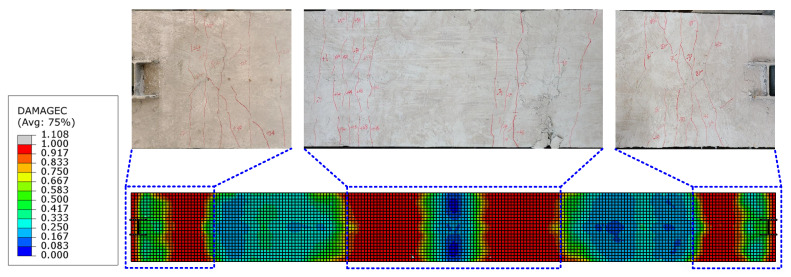
Comparison of the floor slab damage between the test and FEA results.

**Figure 18:**
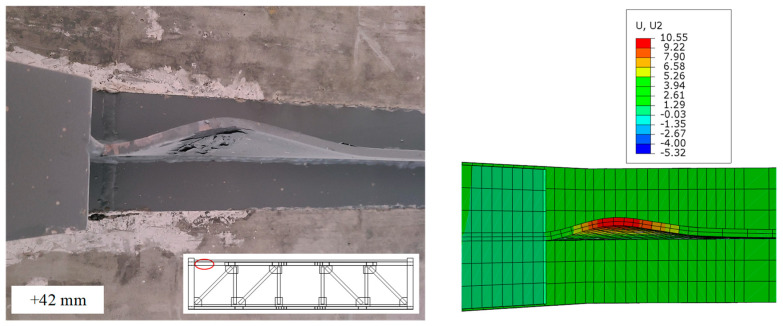
Comparison of the T-shaped member deformation between the test and FEA.

**Figure 19:**
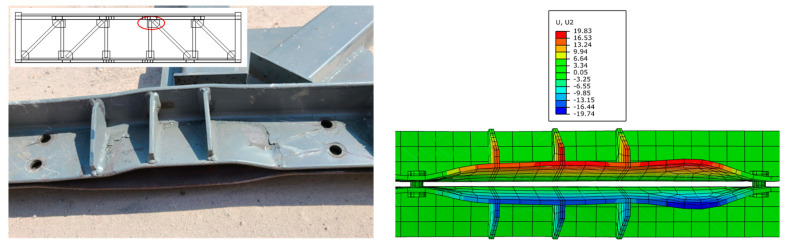
Comparison of the angles deformations between the test and FEA results.

To further validate the effectiveness of the proposed FEM, Figure 17 shows the comparison of experimental floor slab damage and numerical simulation results after loading is completed. The results indicate that the numerical results are in good agreement with the experimental results. The damage model of concrete can effectively simulate the damage of floor slabs. Figure 18 compares the buckling of the T-shaped member at the end of the top chord between experimental and finite element analysis, while Figure 19 compares the deformation of the angles after the end of the experiment. The results show that the deformation of the specimen in the FE model can be matched well with the experimental results.

Overall, the proposed FEM could predict the response of the frictional energy dissipation steel truss subjected to lateral load because it accurately captured the deformation behavior of FED-ST40 and the key points of the load-displacement curves, such as the initial lateral stiffness, the peak, and the ultimate load and displacement.

**Figure 20:**
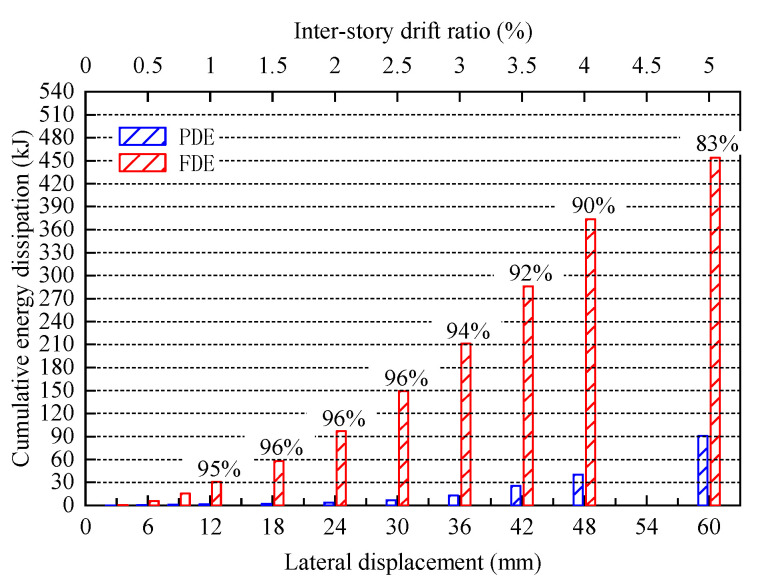
Comparison of frictional dissipated energy (FDE) and plastic dissipated energy (PDE) in FEA.

In addition, different types of energy dissipation can be extracted from the FEM results; the comparison of frictional dissipated energy (FDE) and plastic dissipated energy (PDE) under different loading displacements is shown in Figure 20. In the friction stage, the proportion of FDE has always been greater than 92%, in the plastic stage (42 mm–60 mm), due to significant plastic deformation of the specimen, the proportion of plastic energy dissipation has increased. The final proportion of FDE is 83%, which is far greater than the plastic energy dissipation. This indicates that friction dampers can greatly improve the energy dissipation capacity of the structures.

### 5.4. Effect of Cover Plate Width

**Figure 21:**
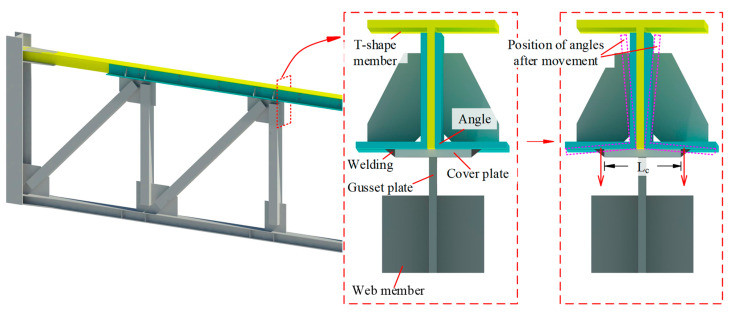
Connection details of the top chord and web members.

The connection details of the top chord and web members are shown in Figure 21. The webs are connected to the angles through a cover plate, which is connected to the angles through two welding seams. The width of the cover plate is $L_{c}$. When the web member applies a downward tensile force on the frictional composite chord, due to the force point of the angles being located at the weld seams, the angles will twist in the section without bolts, forming an “opening” deformation mode as shown in Figure 19, which has adverse effects on the bolt pre-tightening force and the flexural strength of the frictional chord. This unfavorable deformation can be improved by reducing the width of the cover plate; therefore, numerical models FE-CP16, FE-CP30, and FE-CP45 were established to compare with the experimental model FE-Test. The difference between FE-CP16, FE-CP30, FE-CP45, and FE-Test is only that the width of the cover plate has changed from 60 mm of the test specimen to 16 mm, 30 mm, and 45 mm. 

**Figure 22:**
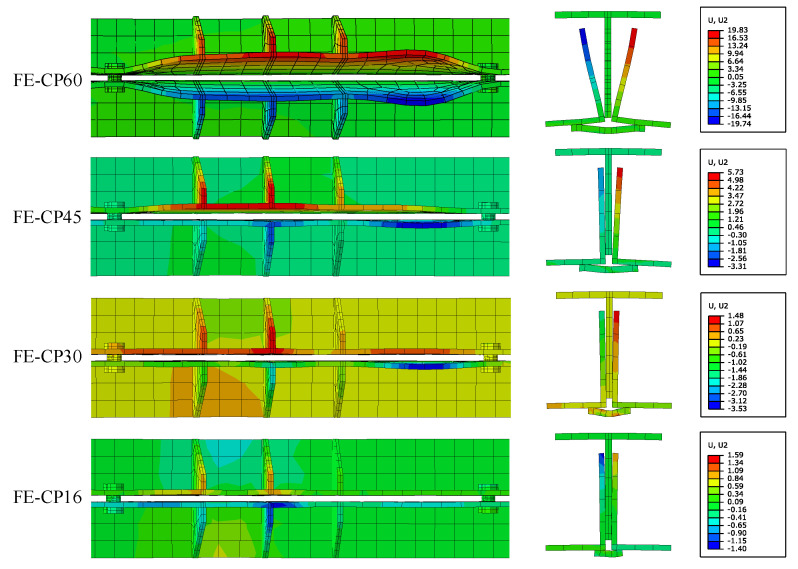
The FEA results of angle members’ final deformations.

**Figure 23:**
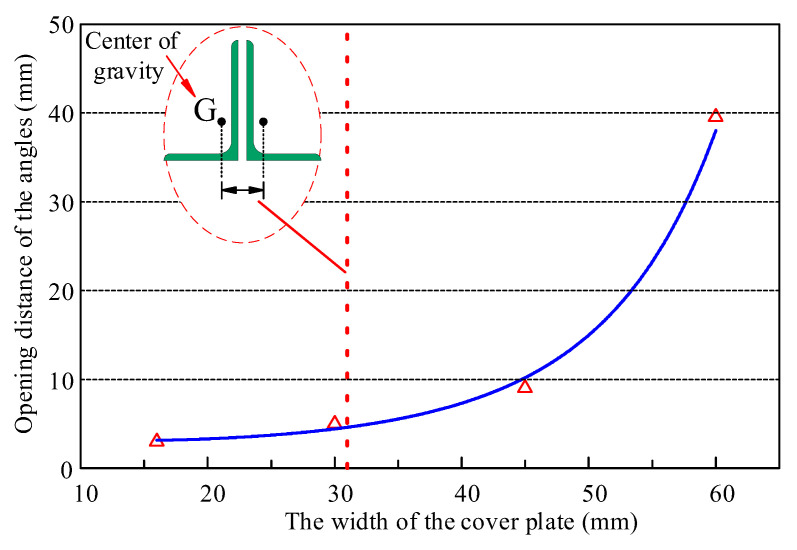
Opening distance of angle members with different cover plate width.

The FEA results of angle members’ deformations are shown in Figure 22 and Figure 23. The smaller the width of the cover plate, the smaller the angles’ deformations. The use of narrow cover plates can effectively control the torsional bulking of angles. In addition, when the width of the cover plate is less than the distance between the centers of gravity of the two angles (31 mm), the deformation of the angles is significantly reduced. Therefore, it is recommended that the width of the cover plates be less than the distance between the centers of gravity of the two angles during design.

## 6. Conclusions

1. The seismic behavior of the FED-ST has been experimentally investigated on a 1:2.5-scale specimen. The experiment verified that the FED-ST showed the expected failure mode and expected three-stage behavior under lateral forces. The ultimate lateral displacement of the FED-ST specimen is 2.6 times that of the traditional truss specimen, the ductility coefficient is 6.9 times that of the traditional truss specimen, and the cumulative energy dissipation is 7.3 times that of the conventional truss specimen. The equivalent viscous damping coefficient of the FED-ST specimen remained at a high level during the test. FED-ST has better energy dissipation capacity and ductility compared to conventional trusses, which will greatly improve the seismic behavior of the STFS. The steel used in this experiment is Q355 steel, while the seismic performance of trusses made of other types of steel needs further research.
2. The proposed FE model was able to predict the response of the frictional energy dissipation steel truss subjected to lateral load based on the deformation behavior of the FED-ST and key characteristics of the load-displacement curves. Therefore, it is advised that it be applied in actual practice for structural design and analysis.
3. The parametrical numerical analyses reveal that the smaller the width of the cover plate connecting the web members and the angle members, the smaller the angles’ deformations. The use of narrow cover plates can effectively control the torsional bulking of angles. Moreover, when the width of the cover plate is less than the distance of the centers of mass of the two angles, the deformation of the angles is significantly reduced. Therefore, it is recommended that the width of the cover plates be less than the distance of the centers of mass of the two angles during design.

## Figures and Tables

**Table 1:** Material properties of the specimen.

Component	Material	Steel Thickness	$f_{\mathrm{cu}}$/MPa	$f_{\mathrm{cu},k}$/MPa	Yield Strength $f_{y}$/MPa	Ultimate Strength $f_{u}$/MPa
Slabs	Concrete C30	-	34.8	31.4	-	-
Chords	Steel Q355	5 mm	-	-	407	589
Webs		3.2 mm	-	-	407	551

**Table 2:** Key parameter values for concrete in ABAQUS.

Dilatation Angle	Eccentricity Ratio	fb0 /fc0	K	Viscosity Coefficient
40	0.1	1.225	0.6667	0

**Table 3:** Key parameter values for steel in ABAQUS.

Es	Poisson’s Ratio	Kinematic Hard Parameters $C_{1}$	Change Ratio of the Back Stress $\gamma_{1}$	$Q_{\infty }$	Hardening Parameter *b*
206,000	0.3	7500	50	$0.5f_{y}$	0.1

## Data Availability

Data will be made available on request.
